# Design and Growth of Low Resistivity P-Type AlGaN Superlattice Structure

**DOI:** 10.3390/mi15050596

**Published:** 2024-04-29

**Authors:** Yang Liu, Xiaowei Zhou, Peixian Li, Bo Yang, Zhuang Zhao

**Affiliations:** 1 School of Advanced Materials and Nanotechnology, Xidian University, Xi’an 710071, China; yliu_333@stu.xidian.edu.cn (Y.L.); yangbo1@stu.xidian.edu.cn (B.Y.); 22141214314@stu.xidian.edu.cn (Z.Z.); 2 State Key Discipline Laboratory of Wide Band Gap Semiconductor Technology, Xidian University, Xi’an 710071, China

**Keywords:** superlattice, barrier doping, periodic thickness, low resistivity

## Abstract

This work investigated the impact of periodic thickness and doping region on the doping efficiency of the P-type AlGaN superlattice. In this paper, the band structure of the simulated superlattice was analyzed. The superlattice structure of Al_0.1_Ga_0.3_N/Al_0.4_Ga_0.6_N, and the AlGaN buffer on the sapphire substrate, achieved a resistivity of ~3.3 Ω·cm. The results indicate that barrier doping and low periodic thickness offer significant advantages in introducing a reduction of the resistivity of P-type AlGaN superlattice structures.

## 1. Introduction

Group III nitrides, particularly the Al_x_Ga_1-x_N ternary alloy, exhibit a tunable band gap ranging from 3.4 eV to 6.2 eV with an Al component x = 0~1, corresponding to the ultraviolet luminescence region with wavelengths ranging from 365 nm to 200 nm [1,2,3]. AlGaN-based electronics have great application prospects, especially in deep ultraviolet light-emitting diodes (DUV-LEDs) [4,5] and solar-blind photodetectors [6,7].

Although UV-LED has made a series of advances [8,9], the realization of P-type doping in AlGaN materials as hole injection layers, particularly those with high Al composition such as AlGaN and AlN [10,11], for use in the hole injection area has been a global challenge. Since 1989, H. Mano et al. utilized low-energy electron beam irradiation (LEEBI) to process Mg-doped GaN, achieving low-resistance P-type GaN for the first time [12]. Various methods have been explored, including rapid thermal annealing [13,14], polarization-induced doping [15,16], Mg-δ doping [17,18], and superlattice doping [19,20]. In 2022, Jiaming Wang et al. [18] employed Mg-δ doping in Al_0.5_Ga_0.5_N, achieving a hole concentration of 8.1 × 10^18^ cm^−3^. Even though Mg-δ doping can achieve a higher hole concentration because the metal atoms (Ga and Al) near the surface stop passing through the metal source when Mg-δ is doped [21], the number of Ga-N bond breaks is greater than that of Al-N bonds at high temperatures because the Ga-N bond energy is less than the Al-N bond energy. A large number of Ga vacancies are left near the doped interface. On the one hand, these vacancies are favorable for Mg atoms to enter the AlGaN lattice; on the other hand, a large number of point defects are unfavorable to the crystal quality.

The challenge with Mg-doped AlGaN lies in the deepening acceptor position as the Al component increases, leading to an increase in Mg acceptor activation energy, rising from ~170 meV in GaN to ~670 meV in AlN [10]. The elevated activation energy results in only a small fraction of Mg impurities being thermally activated to generate holes at room temperature. This directly leads to a decrease in hole concentration and an increase in resistivity. Additionally, as the Al component increases, the reduction in AlGaN lattice constant poses challenges for Mg atom incorporation [22]. Typically, p-GaN is employed as the hole injection layer in the fabrication of DUV devices [23]. Despite the ability to introduce a very high concentration of holes, GaN itself possesses a band gap of only 3.4 eV, leading to strong absorption of high-energy photons [24]. Researchers often improve the light extraction efficiency by non-polar AlGaN epilayers [25], inserting distributed Bragg reflectors [26], and photonic crystals [24,27]. Utilizing a p-AlGaN material with an equivalent band gap as the active region can overcome the issue of GaN light absorption, facilitating structural simplification and reducing subsequent packaging steps.

In this work, the Silvaco TCAD software is employed to simulate the incorporation of Mg acceptors in various regions of the AlGaN superlattice structure. We investigate the impact of superlattice structure and period number on the P-type doping efficiency of the AlGaN material. AlGaN superlattice structures with varying compositions were grown on a sapphire substrate using metal-organic chemical vapor deposition (MOCVD). Additionally, an ultraviolet visible light photometer (UV-VIS) was used to analyze the Al composition of the AlGaN superlattice. High-resolution X-ray diffraction (HR-XRD) was used to characterized the superlattice structures. A scanning electron microscope (SEM) was used to analyze cross-sections of the superlattice structures. Furthermore, Hall effect tests were used to analyze the resistivity of the AlGaN superlattice, which can predict the doping efficiency of the superlattice structure.

## 2. Simulation Calculation

**Figure 1:**
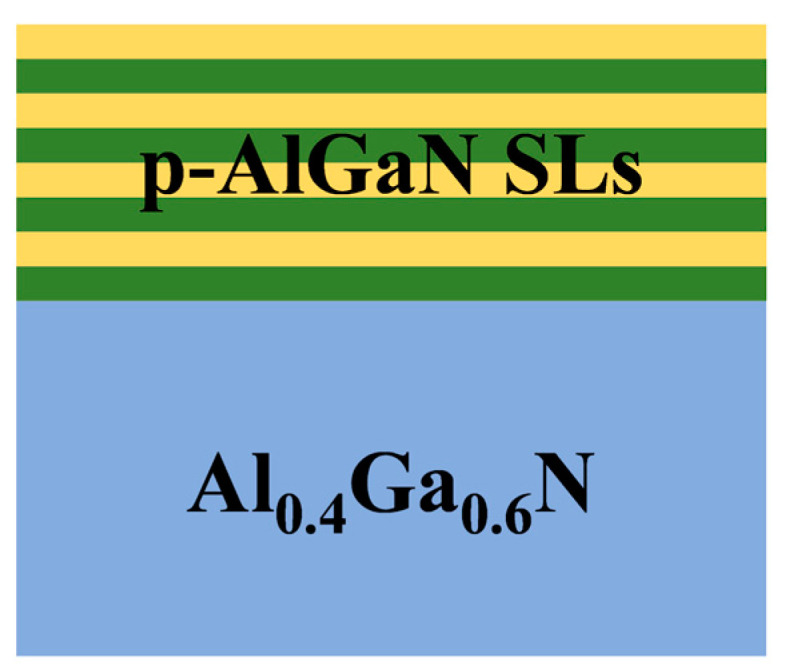
Schematic diagram of Silvaco TCAD simulated superlattice structure.

In this work, the Silvaco TCAD software (2014 version) was used to simulate the AlGaN superlattice structures, varying concentrations of P-type doping, superlattice structure, periodicity, and buffer layer design. The structure diagram of the design is shown in Figure 1.

**Figure 2:**
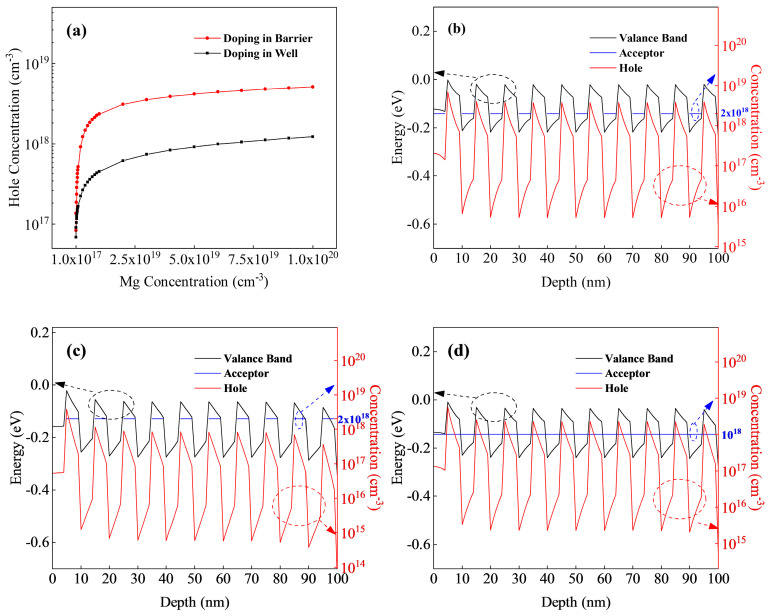
Different doping positions and doses: (**a**) acceptor doping is introduced in different regions of the superlattice structure; (**b**) barrier region doping, acceptor concentration 2 × 10^18^ cm^−3^; (**c**) potential well region doping, acceptor concentration 2 × 10^18^ cm^−3^; (**d**) well barrier co-doping, acceptor concentrations are 1 × 10^18^ cm^−3^.

We investigated the factors influencing the P-type doping efficiency of the superlattice. Figure 2 illustrates the theoretically calculated average hole density in the Mg-doped (5 nm/5 nm) Al_0.1_Ga_0.9_N/Al_0.4_Ga_0.6_N superlattice structure, grown for 10 cycles on the 2 μm Al_0.4_Ga_0.6_ buffer. The residual carrier concentration in the buffer region is set at 1 × 10^14^ cm^−3^. 

In Figure 2a, the red dotted line represents the hole density introduced by the Mg acceptor solely at the barrier layer (Al_0.4_Ga_0.6_N), while the black dotted line depicts the hole density introduced by the Mg acceptor at the potential well layer (Al_0.1_Ga_0.9_N). The hole density introduced by doping at the barrier layer is notably higher than that at the potential well layer under the same doping concentration. In Figure 2b,c, the barrier region and the well region were doped individually, with a doping density of 2 × 10^18^ cm^−3^. Figure 2d illustrates well barrier co-doping with a doping density of 1 × 10^18^ cm^−3^. Given that both the trap layer and the barrier layer have widths of 5 nm, the actual total doping concentration in the superlattice region depicted in Figure 2b–d remains consistent at 1 × 10^18^ cm^−3^. However, the average hole concentration introduced varies, and were 9.1 × 10^17^ cm^−3^, 2.2 × 10^17^ cm^−3^, and 5.5 × 10^17^ cm^−3^, respectively. This indicates that the ionization efficiency of the acceptor is significantly influenced by distinct doping regions, with barrier doping demonstrating a pronounced advantage in hole introduction. As shown in Figure 2b–d, the peak position of hole concentration is at the interface between the potential well and the barrier. The peak concentration of holes in Figure 2b has exceeded the acceptor concentration, indicating that the band structure at the interface strongly affects the acceptor ionization efficiency.

**Figure 3:**
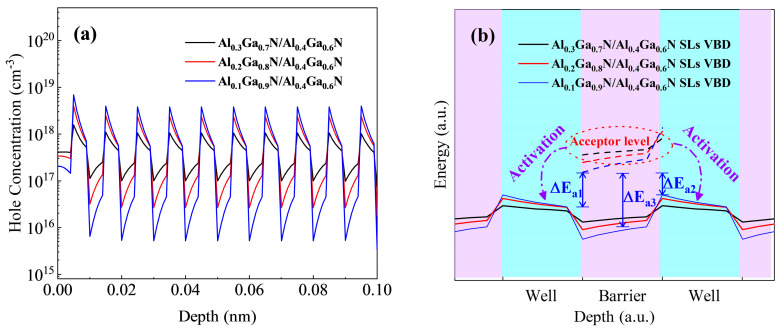
Different Al components in the well layer: (**a**) the change of hole density with location; (**b**) valence band variation and acceptor activation path.

To investigate the impact of superlattice well and barrier Al components on P-type doping, simulation calculations were conducted on a 10-period barrier P-type doped Al_x_Ga_1−x_N/Al_0.4_Ga_0.6_N (x = 0.1, 0.2, 0.3) superlattice grown on a 2 μm Al_0.4_Ga_0.6_N buffer. The doping concentration is 2 × 10^18^ cm^−3^, and the trap and barrier each have a thickness of 5 nm, as illustrated in Figure 3.

As shown in Figure 3a, the three groups of Al_x_Ga_1−x_N/Al_0.4_Ga_0.6_N superlattice structures are doped in the barrier region, and the average hole densities are 9.1 × 10^17^ cm^−3^ (x = 0.1), 6.8 × 10^17^ cm^−3^ (x = 0.2), and 4.4 × 10^17^ cm^−3^ (x = 0.3). The acceptor doping concentration is the same, but the hole concentration is significantly different, as shown in Figure 3b. The main energy level of the barrier region is far from the valence band top of the barrier layer, but because of the existence of the superlattice well region, namely the low Al component AlGaN, the barrier region is easily activated by the main energy level, trapping the valence band top electrons in the potential well region and contributing holes, which is conducive to reducing the ionization energy of the acceptance. From Figure 3a, it can be clearly seen that the larger the Al gap between the well region and the barrier region of the AlGaN superlattice is, the larger the valence band top gap is, and the more favorable it is to superlattice P-type doping.

Additionally, because of the difference in the Al components of the superlattice well and the barrier layer, the average Al component of the superlattice structure changes. Therefore, the superlattice structure of the three groups of samples in Figure 3a is subjected to different stresses in the buffer layer that introduce different piezoelectric polarization electric fields. The Al_0.1/0.4_ superlattice structure with the largest difference in Al components between the potential barrier and the potential well is subjected to greater compressive stress in the buffer layer, which is opposite to the direction of spontaneous polarization of the AlGaN material, promoting the formation of a polarization charge and generating more polarization-induced holes. This polarization is reflected in the band diagram in Figure 3b as a sharp change in the slope of the valence band at the junction of the barrier and the well.

In previous studies, the resistance of superlattice structures in the vertical direction was inversely proportional to the thickness of the structure [18,28]. The resistivity is determined by carrier mobility, carrier concentration, and temperature. We designed our experiment to change the period number of the superlattice structure to study the effect of the period number of the superlattice on the P-type doping efficiency of the AlGaN superlattice, and simulated the 10-, 20-, and 40-period superlattice and found that the period number has little effect on the average hole density. This means that the transport efficiency of the holes in the vertical direction varies with the superlattice period, that is, the more cycles there are, the higher the mobility of the holes in the vertical direction. As stated in the resonant tunneling model proposed by R. Tsu and L. Esaki et al. [29], the barrier layer of the superlattice structure is narrow, and the bound energy levels between each quantum well can be coupled to form microstrips. The greater the number of periods, the wider the microstrip width and the smaller the series resistance.

## 3. Experiment

**Figure 4:**
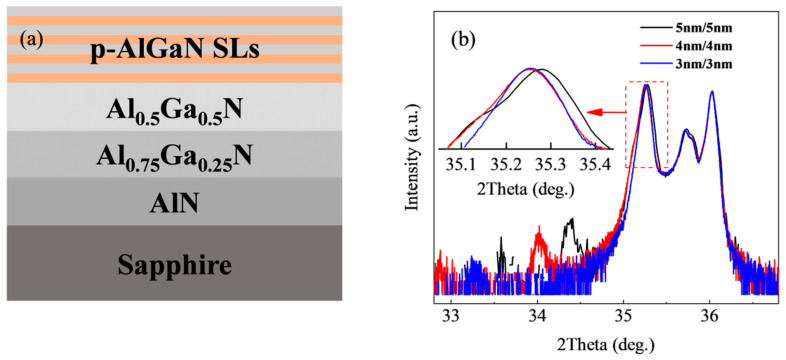
(**a**) Schematic illustration of the sample; (**b**) HR-XRD diffraction pattern scanned by 2theta-omega on the surface (0002) of the superlattice sample with different period thicknesses, 3 nm/3 nm, 4 nm/4 nm, and 5 nm/5 nm.

All samples were grown on 2 in sapphire substrates using Metal-Organic Chemical Vapor Deposition (MOCVD) equipment (AIXTRON CRIUS 2, Herzogenrath, Germany), and TMAl, TMGa, and NH_3_ were used as the sources, with H_2_ as the carrier gas. Firstly, the low temperature AlN nucleation layer of 20 nm was grown at 620 °C. Secondly, the temperature of the reaction chamber is raised to 1080 °C to grow 500 nm AlN and 500 nm Al0._75_Ga0._25_N as the transition layer. Then, 1.5 μm of AlN buffer is grown by epitaxy. Finally, the superlattice structure is grown. Only superlattice barrier P-type doping was performed, using Cp_2_Mg as the dopant with a flux of 280 sccm. The superlattice structure was controlled in 10 cycles. After the epitaxy was completed, each sample was annealed in a N_2_ atmosphere at 850 °C for 10 min and regional information is detailed in Table 1. The only difference among samples A, B, and C lies in the period thicknesses, which were 6 nm, 8 nm, and 10 nm, respectively; the only difference among samples C, D, and E lies in the Al composition of the well layers, which were 0.3, 0.2, and 0.1, respectively. Figure 4a is a schematic diagram of the sample.

Figure 4b shows the superlattice structure with a designed barrier and well thickness of 4 nm, and the diffraction peak and superlattice satellite peak of the buffer layer are clearly visible. The designed superlattice structure has an average Al composition of 0.25, and the superlattice 0-level satellite peak is expected to be near 35.07°. Additionally, the 2theta distance of the superlattice satellite peak near 32.95° and 34.02° is 1.12°, providing evidence for the existence of a superlattice 0-level satellite peak near 35.1°. Since the total thickness of the grown superlattice structure is ~80 nm, it is too thin compared with the ~1.5 μm of Al_0.5_Ga_0.5_N buffer, so the 0-level peak signal of the superlattice is weaker and is covered by the signal of the buffer layer.

In order to verify this conjecture, we adjusted the periodic thickness of the superlattice layer, as shown in Figure 4b. The illustration of Figure 4b shows that the 2theta range is 35.05° to 35.45°. It can be seen that on the left side of the Buffer1 peak, the broadening intensifies as the thickness of the superlattice increases, and the superlattice 0-level peak signal becomes stronger. However, there is a broadening on the right side of the Buffer2 diffraction peak in all three samples, and the signal only increases slightly when the superlattice period thickness is 10 nm and the position does not change with the superlattice period thickness. It is speculated that the junction between AlN and Al_0.75_Ga_0.25_N grows on the substrate and Ga atoms and Al atoms diffuse toward each other to form a gradient layer of Al components between 0.75 and 1, which is called Buffer3. This broadening has existed in previous studies but has not been described in detail [30,31].

**Figure 5:**
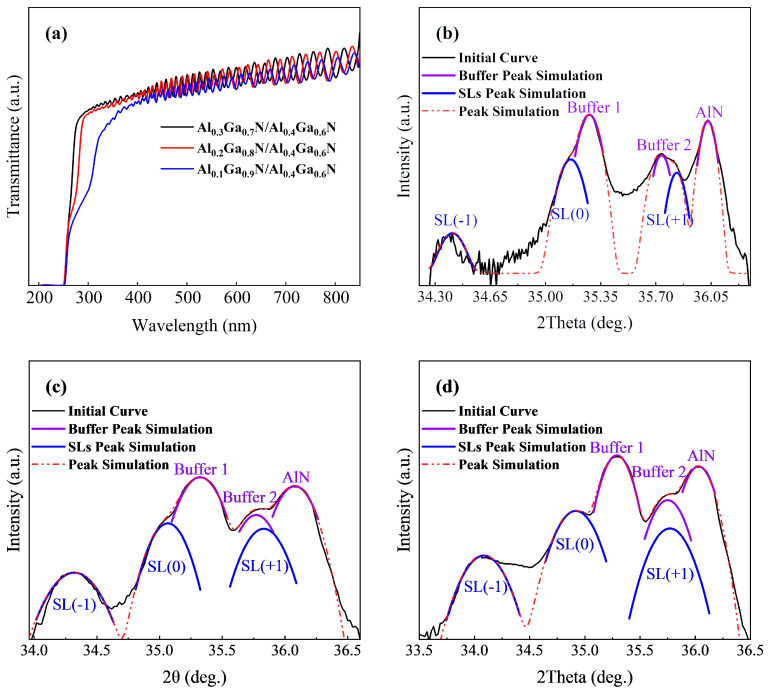
(**a**) UV-VIS transmittance curves of the three groups of samples; (**b**–**d**) HR-XRD (0002) 2theta-omega scanning fitting curves of the three samples.

In order to study the source of the signal change, we grew Al_x_Ga_1 − x_N/Al_0.4_Ga_0.6_N superlattice structures with Al components x = 0.1 and 0.2 and potential well and barrier thickness of 5 nm on the same buffer structure, as shown in Figure 5. 

In Figure 5a, the transmittance curves of the three sample groups exhibit significant absorption starting at 280 nm, 290 nm, and 305 nm. The corresponding Al compositions are 0.36, 0.31, and 0.24, closely matching the average Al components of the designed superlattice structure (0.35, 0.31, and 0.24). The thickness of the superlattice region of all samples is ~100 nm, and the change of the Al component in the superlattice structure Al_x_Ga_1 − x_N/Al_0.4_Ga_0.6_N has little effect on the change of the Al component in the whole sample. However, in the band below 305 nm, all samples have a strong absorption effect, indicating that in order to reduce the light absorption of the DUV-LED p-layer, it is necessary to increase the Al component in P-type region.

The XRD diffraction of the superlattice follows the Bragg equation [32,33]:

2Dsinθ_L_ = Lλ, (1)

In Equation (1), D is the periodic thickness of the superlattice structure, L is the order of the satellite peak in 2θ-ω scanning, and θ_L_ is the Bragg angle corresponding to the satellite peak. The superlattice structure has symmetric diffraction. For HR-XRD diffraction of the superlattice, the corresponding distance Δθ between two adjacent satellite peaks is expressed by Equation (2):

(2) $$\Delta \theta =\frac{\lambda }{2\mathrm{Dcos}\overset{-}{\theta }}\Delta L$$

In Equation (2),$\overset{-}{\theta }$ is the average Bragg angle of the two satellite peaks. Let ΔL = 1, then the superlattice structure thickness can be approximated as:

(3) $$D=\frac{\lambda }{2\Delta \theta \cos\overset{-}{\theta }}$$

According to Equation (3), the scanning pattern of the HR-XRD (0002) 2θ-ω superlattice structure in Figure 4 is processed, and the satellite peak position is obtained, as shown in Table 2.

In Figure 5b–d, the positions of the superlattice satellite peaks in the three sample groups shift to the left as the Al component decreases, while the average peak spacing (theta) remains approximately 0.35°. Following peak splitting simulation, depicted in Figure 5b–d, when the Al component of the well layer decreases, the average Al component of the superlattice structure also decreases. This leads to an increase in compressive stress in the superlattice layer, a decrease in the C-axis lattice constant, a reduction in the (0002) crystal plane spacing, and a leftward shift in the satellite peak position of the superlattice. However, since the periodic thickness remains constant, the distance between superlattice satellites also remains constant. Consequently, the position of the superlattice satellite peak SL(+1) in Figure 4b precisely coincides with the signal position caused by Buffer3. In contrast, the superlattice structures with periodic thicknesses of 8 nm and 6 nm in Figure 3 appear relatively small due to their reduced periodic thickness. The position of the SL(+1) peak in the gradient region does not impact its signal due to its specific position. Consequently, the signal at the Buffer3 position in Figure 3b only exhibits a slight elevation in the Al_0.3_Ga_0.4_N superlattice structure with a periodic thickness of 10 nm. All the peak information is shown in Table 2. 

The hole concentration in the AlGaN material can be expressed by the following formula [22]:

(4) $$P=\frac{\left(N_{A}-N_{D}\right)}{1+g_{p}/N_{v}exp(\Delta E_{A}/kT)}$$

In Equation (4), N_A_ is the acceptor concentration of AlGaN, N_D_ is the donor concentration, g_p_ is the degeneracy factor, N_V_ is the valence-capped effective state density, and E_A_ is the acceptor activation energy.

As shown in Equation (4), the hole concentration P is a function of the net acceptor doping concentration (N_A_ − N_D_) and the acceptor element activation energy (E_A_). The relationship between hole concentration and net acceptor doping concentration is linear, while the relationship between hole concentration and acceptor activation energy is a negative exponential. Therefore, the hole concentration depends mainly on the acceptor activation energy. The activation energy of Al_0.27_Ga_0.73_N was 0.31 eV by Mg doping [34]. At room temperature, the same net acceptor concentration was maintained, the Mg acceptor activation energy E_A_ = 0.31 eV in Al_0.27_Ga_0.73_N was reduced to E_A_ = 0.17 eV in GaN, and the hole concentration was increased by ~1 order of magnitude. Superlattice structures make this possible. Due to the different bandgap widths of superlattice materials, the energy bands at the interface of the two materials will mutate, and the conduction band and valence band will produce the same periodic oscillation of the superlattice period [35]. The acceptances at the interface between the potential well and the potential barrier will undergo dissociation, and the holes generated by the dissociation will occupy the edge of the valence band far away from the Fermi level, forming a periodic distribution of quasi-two-dimensional hole gas. Although this void gas is not continuous, its average void concentration still exceeds the doping in the bulk material, as shown in Figure 1.

Room temperature Hall effect tests were performed on all samples, and Table 2 illustrates that when the thickness of the superlattice trap and barrier decreases (sample A and sample B), it leads to a decrease in resistivity. This is due to the increase in energy band oscillation amplitude as the periodic thickness decreases. The acceptor energy level of the barrier layer decreases in relation to the valence band top position of the well layer, making the acceptor more easily ionized. This results in a decrease in the acceptor activation energy, promoting an increase in hole density. Consequently, when the periodic thickness of the superlattice is reduced from 12.3 nm to 5.2 nm, the resistivity decreases by approximately one order of magnitude. When the superlattice period thickness remains constant, reducing the Al component of the well layer also contributes to an increase in hole density and a decrease in resistivity. Because of the presence of the low Al component well layer, the deep activation path of the barrier layer becomes shorter, making it easier to be ionized, as depicted in Figure 2b. 

In Table 2, the Al component of AlGaN buffer structure changes from 1 to 0.5, Al_0.5_Ga_0.5_N of ~1.5 μm is grown, and the total thickness of all superlattice structures is ~100 nm. The buffer provides compressive stress to the superlattice structure and is responsible for inducing the formation of holes. For samples A, B, and C, the lattice constants between the barrier and the potential well are close to each other, and a slowly changing region is easily formed, which is similar to the growth of a layer of the Al component of the ~0.35 AlGaN material on the Al_0.5_Ga_0.5_N buffer. On the whole, the compressive stresses are smaller than that of samples D and E, and the hole densities induced by polarization are smaller than that of samples D and E. However, because the periodic thicknesses of samples A and B are small and the valence band changes more steeply, which is conducive to hole transmission, the resistivities of samples A and B are much smaller than that of sample C. However, the Al components in the potential barrier and potential well regions of the superlattice structure of samples D and E differ greatly, and the average Al components are lower. Therefore, the polarization induced holes and the Mg acceptances work together to make samples D and E have higher hole densities. At the same time, the band gaps of samples D and E are very different; the band at the junction of the potential well and the barrier changes, forming a conductive channel near the potential well, and the holes are transported in the form of two-dimensional hole gas, resulting in the low resistivities of samples D and E.

**Figure 6:**
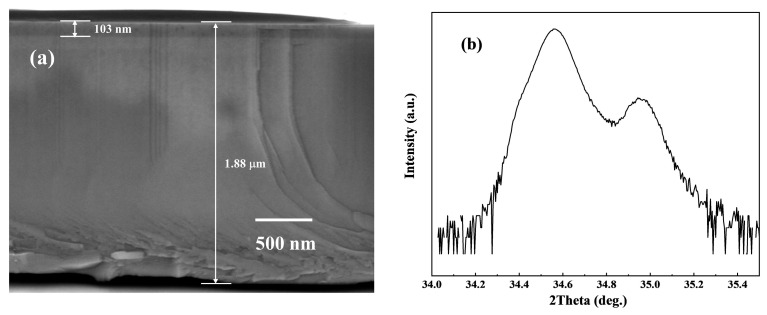
(**a**) Cross-section of growing AlGaN superlattice on a GaN buffer; (**b**) HR-XRD (0002) 2theta-omega scanning fitting curves of sample F.

In order to verify the effect of polarization induction and acceptor doping on the AlGaN superlattice, we grow an AlGaN superlattice sample F with the same structure as sample E on a GaN buffer. The SEM and HR-XRD scanning patterns of its cross-sections are shown in Figure 6.

As shown in Figure 6a, the superlattice structure cannot be seen in the SEM scanning image. In Figure 6b, in the HR-XRD 2θ-ω scan of sample F, the satellite peak of the superlattice structure cannot be seen. Only the GaN buffer peak around 34.57°, the AlGaN peak around 34.95°, and some miscellaneous peaks can be seen. As calculated from Equation (1), the component of Al is 0.27. The superlattice structure cannot be seen in the SEM scanning image. This phenomenon is due to the fact that the buffer layer uses a GaN with a large lattice constant to exert tensile stress on the upper AlGaN structure, resulting in the insufficient periodic transition of the superlattice structure; the small periodic thickness of the superlattice structure is also the main factor.

The hall effect of sample F was tested at room temperature and its resistivity was found to be ~209 Ω·cm, which is two orders of magnitude higher than that of sample E. It is about three times higher than the sample C with the highest resistivity on the AlGaN buffer. Such a high resistivity difference is due to the different types of stress on the superlattice structure caused by different buffer materials. Empirically, we can view the superlattice as the bulk material of strain and the buffer layer as the bulk material of relaxation. Then the superlattice structure is subjected to both piezoelectric polarization and spontaneous polarization, and only spontaneous polarization exists in the buffer layer. The difference of polarization intensity near the interface is the polarization surface charge caused by the change of polarization intensity. As shown in Table 2, we know the average Al component in AlGaN, and according to the polarization effect formula [36], we calculated the tensile stress generated by the GaN buffer on the upper AlGaN, and compressive stress by the Al_0.5_Ga_0.5_N buffer on the upper AlGaN SLs.

All the samples grown on the AlGaN buffer have Al components smaller than or equal to the buffer layer, which ensures that the superlattice structure is subjected to compressive stress. While compressive stress is conducive to improving the crystal quality of the upper superlattice, it also generates a piezoelectric polarization electric field due to piezoelectric polarization. The piezoelectric polarized electric field changes the band structure of the superlattice and induces polarization-induced holes, which is beneficial to increase the hole concentration of the superlattice structure and reduce the resistivity. In sample F, the buffer layer exerts tensile stress when growing the superlattice structure, which is not conducive to material growth. The polarization-induced electrons produced by AlGaN under tensile stress will passivate the doped acceptor impurities, which is not conducive to the reduction of resistivity.

## 4. Discussion

Reducing the thickness of the superlattice barrier layer and increasing the difference of Al components between the superlattice well and the barrier are beneficial to reducing the resistivity. The reason is that reducing the thickness is conducive to increasing the probability of tunneling in the hole band, and increasing the difference of Al components is conducive to band mutation and reducing the activation energy of the acceptor. Both of these methods are beneficial to reduce the longitudinal resistance of the superlattice structure.

The realization of a low resistivity P-type AlGaN superlattice structure is not only beneficial to reduce the light absorption of LED devices and improve the output power, but also has broad prospects for field effect tubes. Using a polarization-induced two-dimensional electron gas at the AlGaN/GaN heterojunction interface, HEMT devices have developed rapidly, while complementary P-type transistors have developed more slowly. P-type doping in the AlGaN superlattice barrier layer is conducive to increasing the concentration of holes, and the high barrier between the potential well layers is conducive to the transverse transport of holes in the potential well, forming a two-dimensional hole gas transport form. Therefore, it is necessary to reduce the tunneling in the hole zone. We can increase the hole concentration by increasing the thickness of the barrier layer and the difference of Al components between the barrier and the potential well, and thus increase the hole transport in the well as much as possible. This has an obvious promoting effect on the development of AlGaN-based field effect transistors.

The stress effect of the buffer layer on the superlattice structure is also an important factor affecting the doping efficiency. Therefore, the selection of higher quality sapphire/AlGaN templates or single crystal AlN as substrates is another choice to achieve low resistivity p-AlGaN superlattices.

## 5. Conclusions

In this study, Silvaco TCAD is employed to investigate the energy band structure and doping characteristics of p-AlGaN superlattices with varying components and periodic thicknesses. The results indicate that different doping regions significantly impact the ionization efficiency of the acceptor, with barrier doping having a greater advantage in introducing holes. The superlattice structure was characterized using HR-XRD and UV-VIS, while the doping properties were assessed through Hall effect tests. Adding Mg to the Al_0.1_Ga_0.4_N/Al_0.4_Ga_0.6_N superlattice barrier with a single period thickness of 10 nm for 10 periods results in a resistivity of ~3.3 Ω·cm. These values align with the theoretical analysis.

## Figures and Tables

**Table 1:** Information on potential wells and barriers of superlattice structures.

Sample		A	B	C	D	E
Well	Al Composition	0.3	0.3	0.3	0.2	0.1
	Thickness (nm)	3	4	5	5	5
Barrier	Al Composition	0.4	0.4	0.4	0.4	0.4
	Thickness (nm)	3	4	5	5	5

**Table 2:** HR-XRD scanning 2θ peak information of all samples.

Sample	A	B	C	D	E
SLs(−1)	33.3°	34.02°	34.39°	34.27°	34.08°
SLs(0)	35.09°	35.09°	35.10°	35.01°	34.92°
SLs(+1)	/	/	35.82°	35.78°	35.75°
Buffer 1	35.26°	35.25°	35.27°	35.28°	35.29°
Buffer 2	35.72°	35.74°	35.73°	35.72°	35.75°
AlN	36.03°	36.02°	36.03°	36.03°	36.03°
SLs Al Composition	0.36	0.36	0.37	0.31	0.25
SLs Periodic thickness (nm)	5.2	8.8	12.3	12.5	11.0
Resistivity (Ω·cm)	6.2	23.7	66.2	16.1	3.3

## Data Availability

All data that support the findings of this study are included within the article.
